# Crystal structure of vilazodone hydro­chloride methanol monosolvate

**DOI:** 10.1107/S2056989016017734

**Published:** 2016-11-10

**Authors:** Xiu-Rong Hu, Jia-Li Ye, Jian-Ming Gu

**Affiliations:** a Chemistry Department, Zhejiang University, Hangzhou 310028, People’s Republic of China; b College of Pharmaceutical Science, Zhejiang Chinese Medical University, Hangzhou 310053, People’s Republic of China

**Keywords:** crystal structure, benzo­furan, indole, piperazine, hydrogen bonds

## Abstract

In the title compound, C_26_H_28_N_5_O_2_^+^·Cl^−^·CH_3_OH {systematic name: 4-(2-carbamoyl-1-benzo­furan-5-yl)-1-[4-(5-cyano-1*H*-indol-3-yl)but­yl]piperazin-1-ium chloride methanol monosolvate}, the protonated piperazine ring adopts a chair conformation. The indole ring plane is nearly perpendicular to the benzo­furan ring system, with a dihedral angle of 85.77 (2)°. In the crystal, the organic cations, Cl^−^ anions and methanol solvent mol­ecules are linked by classical N—H⋯O and N—H⋯Cl hydrogen bonds, and weak C—H⋯O and C—H⋯π inter­actions into a three-dimensional supra­molecular architecture.

## Abstract 2

In the title compound, the protonated piperazine ring adopts a chair conformation while the indole ring plane is nearly perpendicular to the benzo­furan ring system.

## Chemical context   

Major depression disorder (MDD) currently ranks as the world’s fourth greatest cause of illness and is expected to rank second by the year 2020 according to WHO studies (Murray & Lopez, 1996 ▸). The title compound, viladozone hydro­chloride (marketed as Viibryd by Forest Pharmaceuticals), is a new treatment option for MDD. It was approved on January 21, 2011 by the FDA, licensed by Merck KGaA.
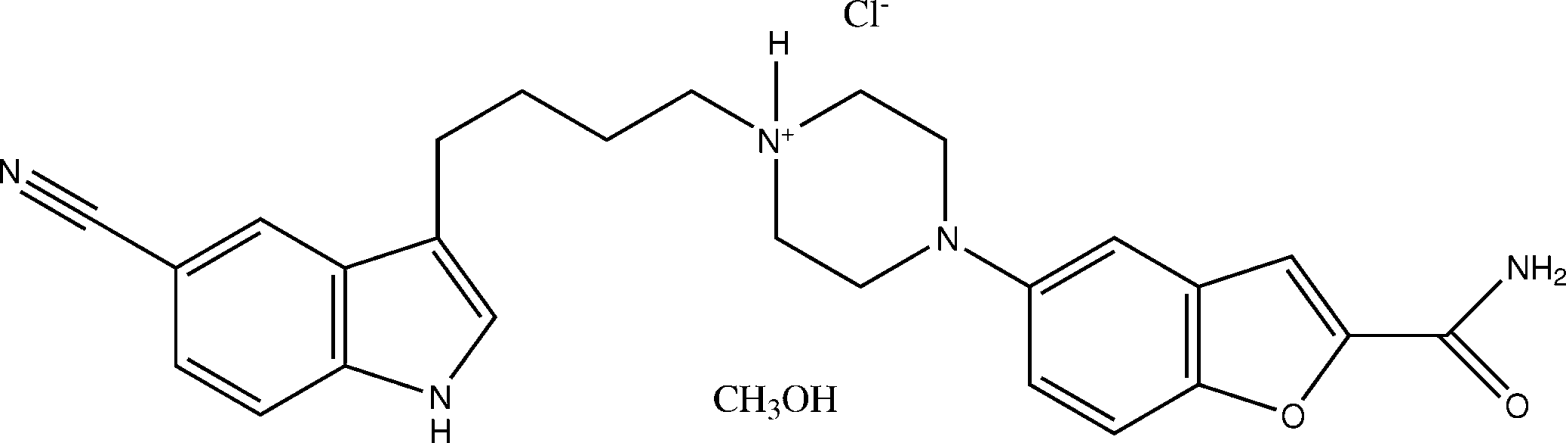


Vilazodone hydro­chloride is a selective serotonin re-uptake inhibitor (SSRI) with properties that are most similar to those of citalopram, escitalopram (levapro), fluoveline, proxetin, and sertraline. The new drug differs from its predecessors by also acting as a partial agonist at serotonergic 5-HT_1A_ receptors. The mechanism of the anti­depressant effect of vilazodone is thought to be related to its enhancement of serotonergic activity in the CNS through selective inhibition of serotonin re-uptake. Vilazodone binds with high affinity to the serotonin re-uptake site but not to the norepinephrine or dopamine re-uptake site. As a result, vilazodone potently and selectively inhibits the re-uptake of serotonin (Choi *et al.*, 2012 ▸; Reed *et al.*, 2012 ▸; Schwartz & Singh, 2012 ▸). Many patents and papers have been reported on the synthesis, polymorphism and bioavailability of this drug (Bathe *et al.*, 2011 ▸; Heinrich & Böttcher, 2004 ▸; Leksic *et al.*, 2013 ▸; Lu *et al.*, 2012 ▸) but up till now, its three-dimensional structure has not been reported. This work concerns the crystal structure of vilazodone hydro­chloride methanol solvate, (I), studied at 275 K.

## Structural commentary   

**Figure 1:**
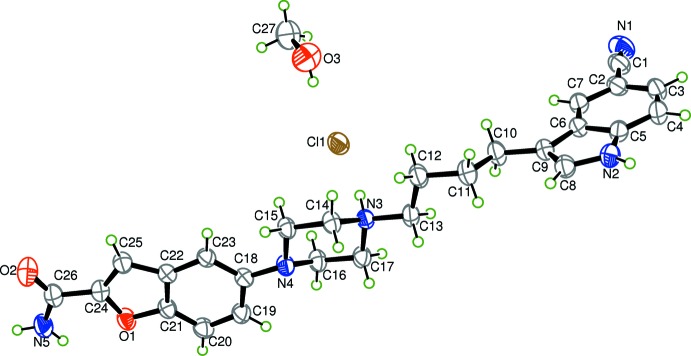
The mol­ecular structure of the title compound, showing the atom-labelling scheme and displacement ellipsoids at 40% probability level. H atoms are shown as small circles of arbitrary radii.

The title compound combines indole-butyl-amine and chromenonyl piperazine structural elements in a single mol­ecular entity. The asymmetric unit of (I) contains one protonated vilazodone cation, one Cl^−^ anion and one methanol mol­ecule (Fig. 1 ▸).

The expected proton transfer from hydro­chloric acid to atom N3 of piperazine occurs; the H atom on the piperazine N3 atom was located unequivocally in the electron-density map. The six-membered piperazine ring adopts a chair conformation. The electron-withdrawing cyano group at position 5 on the indole is twisted out of the mean plane of the indole unit, as indicated by the relevant torsion angles N1—C1—C2—C7 and N1—C1—C2—C3 [144.3 (2) and 34.0 (2)°, respectively]. The conformation of the cyano group is similar to that of other drugs containing nitrile groups, such as bicalutamide and Febuxostat (Hu & Gu, 2005 ▸; Jiang *et al.*, 2011 ▸). The indole moiety is connected by an *n*-butyl linker to the piperazine ring. The conformation of the butyl chain is of some inter­est. Three C atoms of the butyl group (C10, C11 and C12) are coplanar with atom C9 of the indole, as confirmed by the C9—C10—C11—C12 torsion angle of 179.2 (2)°, meanwhile atoms C11, C12 and C13 are coplanar with piperazine atom N3. A dihedral angle of 80.9 (2)° is formed between the mean planes of N3/C11–C13 and C9–C12. The dihedral angle between the C9–C12 mean plane and the indole plane is 10.0 (2)°. The second piperazine N atom, N4, is bonded to the benzo­furan ring. The formamide group is almost coplanar with the connected benzo­furan ring, making a dihedral angle of 2.53 (2)°. The indole ring is almost perpendicular to the benzo­furan ring, as indicated by the dihedral angle of 85.77 (2)° between them.

## Supra­molecular features   

**Figure 2:**
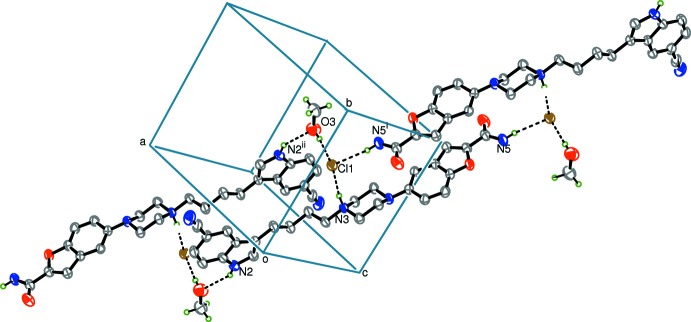
Part of the crystal packing of the title compound. Hydrogen bonds are shown as dashed lines. H atoms not involved in hydrogen bonding have been omitted for clarity.

In the crystal, N3—H3*A*⋯Cl1, O3—H3*B*⋯Cl1 and N2—H2⋯O3^i^ [symmetry code: (i) 1 − *x*, −*y*, 1 − *z*] hydrogen bonds (Table 1 ▸), connect the Cl^−^ ion to two neighbouring cations and a methanol mol­ecule, forming a mol­ecular dimer. Hydrogen bonds N5—H5*A*⋯Cl1^ii^ [symmetry code: (ii) 1 − *x*, 2 − *y*, 2 − *z*] and N3—H3*A*⋯Cl1 link another two neighbouring cations and the Cl^−^ anion into a mol­ecular sheet. As a result, 28-membered rings with the graph-set motif 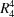
(28) are generated (Fig. 2 ▸).

## Synthesis and crystallization   

Vilazodone hydro­chloride was supplied by Hangzhou HEZE pharmaceutical Technology Co., Ltd. It was recrystallized from methanol solution, giving single crystals suitable for X-ray diffraction.

## Refinement   

Experimental details including the crystal data, data collection and refinement are summarized in Table 2 ▸. The difference density indicated the presence of an H atom at atom N3, showing proton transfer from HCl to the amino group of the vilazodone ring. This H atom was placed in a calculated position with N—H = 0.91 Å and refined as riding with *U*_iso_(H) = 1.2*U*_eq_(N). All other H atoms were placed in calculated positions with O—H = 0.82, N—H = 0.86 and C—H = 0.93–0.98 Å, and included in the refinement in a riding model with *U*_iso_(H) = 1.2 or 1.5*U*_eq_(carrier atom).

## Supplementary Material

Crystal structure: contains datablock(s) global, I. DOI: 10.1107/S2056989016017734/xu5894sup1.cif

Structure factors: contains datablock(s) I. DOI: 10.1107/S2056989016017734/xu5894Isup2.hkl

Supporting information file. DOI: 10.1107/S2056989016017734/xu5894Isup3.cml

CCDC reference: 1439516

Additional supporting information: crystallographic information; 3D view; checkCIF report

## Figures and Tables

**Table 1:** Hydrogen-bond geometry (Å, °) *Cg*5 is the centroid of the C18–C22 ring.

*D*—H⋯*A*	*D*—H	H⋯*A*	*D*⋯*A*	*D*—H⋯*A*
N2—H2⋯O3^i^	0.86	2.25	2.971 (3)	141
N3—H3*A*⋯Cl1	0.91	2.18	3.0787 (19)	172
N5—H5*A*⋯Cl1^ii^	0.86	2.42	3.250 (2)	162
N5—H5*B*⋯N1^iii^	0.86	2.33	3.151 (4)	160
O3—H3*B*⋯Cl1	0.82	2.38	3.195 (2)	171
C13—H13*A*⋯O2^iv^	0.97	2.33	3.272 (3)	164
C19—H19⋯O3^v^	0.93	2.55	3.348 (3)	144
C14—H14*B*⋯*Cg*5^vi^	0.97	2.48	3.393 (2)	156

Symmetry codes: (i) 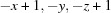; (ii) 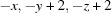; (iii) 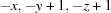; (iv) 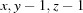; (v) 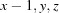; (vi) 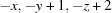.

**Table 2:** Experimental details

Crystal data	
Chemical formula	C_26_H_28_N_5_O_2_^+^·Cl^−^·CH_4_O
*M*_r_	510.03
Crystal system, space group	Triclinic, *P* 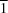
Temperature (K)	296
*a*, *b*, *c* (Å)	10.5572 (5), 11.0764 (4), 11.4408 (5)
α, β, γ (°)	104.622 (1), 97.327 (2), 90.695 (1)
*V* (Å^3^)	1282.57 (10)
*Z*	2
Radiation type	Mo *K*α
μ (mm^−1^)	0.19
Crystal size (mm)	0.50 × 0.46 × 0.38

Data collection	
Diffractometer	Rigaku R-AXIS RAPID/ZJUG
Absorption correction	Multi-scan (*ABSCOR*; Higashi, 1995 ▸)
*T*_min_, *T*_max_	0.91, 0.93
No. of measured, independent and observed [*I* > 2σ(*I*)] reflections	11090, 5000, 3865
*R*_int_	0.044
(sin θ/λ)_max_ (Å^−1^)	0.617

Refinement	
*R*[*F* ^2^ > 2σ(*F* ^2^)], *wR*(*F* ^2^), *S*	0.053, 0.154, 1.00
No. of reflections	5000
No. of parameters	328
H-atom treatment	H-atom parameters constrained
Δρ_max_, Δρ_min_ (e Å^−3^)	0.31, −0.43

Computer programs: *PROCESS-AUTO* (Rigaku, 2006 ▸), *CrystalStructure* (Rigaku, 2007 ▸), *SHELXS97* and *SHELXL97* (Sheldrick, 2008 ▸), *ORTEP-3 for Windows* and *WinGX* (Farrugia, 2012 ▸).

## supplementary crystallographic information

### Crystal data

**Table:**

C_26_H_28_N_5_O_2_^+^·Cl^−^·CH_4_O	*Z* = 2
*M**_r_* = 510.03	*F*(000) = 540
Triclinic, *P*1	*D*_x_ = 1.321 Mg m^−^^3^
Hall symbol: -P 1	Mo *K*α radiation, λ = 0.71073 Å
*a* = 10.5572 (5) Å	Cell parameters from 9260 reflections
*b* = 11.0764 (4) Å	θ = 3.1–27.4°
*c* = 11.4408 (5) Å	µ = 0.19 mm^−^^1^
α = 104.622 (1)°	*T* = 296 K
β = 97.327 (2)°	Chunk, colorless
γ = 90.695 (1)°	0.50 × 0.46 × 0.38 mm
*V* = 1282.57 (10) Å^3^	

### Data collection

**Table:**

Rigaku R-AXIS RAPID/ZJUG diffractometer	5000 independent reflections
Radiation source: rotating anode	3865 reflections with *I* > 2σ(*I*)
Graphite monochromator	*R*_int_ = 0.044
Detector resolution: 10.00 pixels mm^-1^	θ_max_ = 26.0°, θ_min_ = 3.1°
ω scans	*h* = −12→13
Absorption correction: multi-scan (ABSCOR; Higashi, 1995)	*k* = −13→13
*T*_min_ = 0.91, *T*_max_ = 0.93	*l* = −14→14
11090 measured reflections	

### Refinement

**Table:**

Refinement on *F*^2^	Secondary atom site location: difference Fourier map
Least-squares matrix: full	Hydrogen site location: inferred from neighbouring sites
*R*[*F*^2^ > 2σ(*F*^2^)] = 0.053	H-atom parameters constrained
*wR*(*F*^2^) = 0.154	*w* = 1/[σ^2^(*F*_o_^2^) + (0.0589*P*)^2^ + 0.9975*P*] where *P* = (*F*_o_^2^ + 2*F*_c_^2^)/3
*S* = 1.00	(Δ/σ)_max_ < 0.001
5000 reflections	Δρ_max_ = 0.31 e Å^−^^3^
328 parameters	Δρ_min_ = −0.43 e Å^−^^3^
0 restraints	Extinction correction: SHELXL97 (Sheldrick, 2008), Fc^*^=kFc[1+0.001xFc^2^λ^3^/sin(2θ)]^-1/4^
Primary atom site location: structure-invariant direct methods	Extinction coefficient: 0.096 (6)

### Special details

**Table:**

Geometry. All esds (except the esd in the dihedral angle between two l.s. planes) are estimated using the full covariance matrix. The cell esds are taken into account individually in the estimation of esds in distances, angles and torsion angles; correlations between esds in cell parameters are only used when they are defined by crystal symmetry. An approximate (isotropic) treatment of cell esds is used for estimating esds involving l.s. planes.
Refinement. Refinement of F^2^ against ALL reflections. The weighted R-factor wR and goodness of fit S are based on F^2^, conventional R-factors R are based on F, with F set to zero for negative F^2^. The threshold expression of F^2^ > 2sigma(F^2^) is used only for calculating R-factors(gt) etc. and is not relevant to the choice of reflections for refinement. R-factors based on F^2^ are statistically about twice as large as those based on F, and R- factors based on ALL data will be even larger.

### Fractional atomic coordinates and isotropic or equivalent isotropic displacement parameters (Å^2^)

**Table:**

	*x*	*y*	*z*	*U*_iso_*/*U*_eq_	
C1	0.4328 (3)	−0.1560 (3)	−0.0343 (3)	0.0527 (7)	
C2	0.4157 (2)	−0.2028 (2)	0.0690 (2)	0.0456 (6)	
C3	0.4277 (3)	−0.3323 (3)	0.0575 (3)	0.0540 (7)	
H3	0.4489	−0.3833	−0.0146	0.065*	
C4	0.4084 (3)	−0.3835 (2)	0.1517 (3)	0.0523 (6)	
H4	0.4165	−0.4684	0.1448	0.063*	
C5	0.3764 (2)	−0.3039 (2)	0.2580 (2)	0.0428 (6)	
C6	0.3641 (2)	−0.1733 (2)	0.2719 (2)	0.0386 (5)	
C7	0.3851 (2)	−0.1233 (2)	0.1752 (2)	0.0422 (5)	
H7	0.3788	−0.0382	0.1819	0.051*	
C8	0.3290 (2)	−0.2199 (2)	0.4438 (2)	0.0454 (6)	
H8	0.3117	−0.2136	0.5227	0.054*	
C9	0.3331 (2)	−0.1218 (2)	0.3923 (2)	0.0400 (5)	
C10	0.3143 (3)	0.0136 (2)	0.4511 (2)	0.0467 (6)	
H10A	0.2362	0.0387	0.4109	0.056*	
H10B	0.3847	0.0639	0.4386	0.056*	
C11	0.3065 (3)	0.0404 (2)	0.5869 (2)	0.0473 (6)	
H11A	0.3841	0.0139	0.6265	0.057*	
H11B	0.2355	−0.0095	0.5989	0.057*	
C12	0.2892 (3)	0.1776 (2)	0.6494 (2)	0.0483 (6)	
H12A	0.3173	0.1928	0.7362	0.058*	
H12B	0.3420	0.2305	0.6174	0.058*	
C13	0.1509 (2)	0.2113 (2)	0.6295 (2)	0.0424 (5)	
H13A	0.1223	0.1917	0.5425	0.051*	
H13B	0.0993	0.1597	0.6640	0.051*	
C14	0.1686 (2)	0.3853 (2)	0.8187 (2)	0.0401 (5)	
H14A	0.2599	0.3756	0.8351	0.048*	
H14B	0.1254	0.3311	0.8572	0.048*	
C15	0.1392 (2)	0.5194 (2)	0.8736 (2)	0.0390 (5)	
H15A	0.1624	0.5392	0.9614	0.047*	
H15B	0.1901	0.5744	0.8422	0.047*	
C16	−0.0349 (2)	0.5071 (2)	0.7125 (2)	0.0413 (5)	
H16A	0.0126	0.5600	0.6760	0.050*	
H16B	−0.1251	0.5208	0.6952	0.050*	
C17	−0.0103 (2)	0.3716 (2)	0.6576 (2)	0.0429 (5)	
H17A	−0.0620	0.3186	0.6905	0.051*	
H17B	−0.0352	0.3516	0.5700	0.051*	
C18	−0.0372 (2)	0.6568 (2)	0.91099 (19)	0.0355 (5)	
C19	−0.1686 (2)	0.6817 (2)	0.8901 (2)	0.0418 (5)	
H19	−0.2227	0.6231	0.8320	0.050*	
C20	−0.2185 (2)	0.7889 (2)	0.9523 (2)	0.0445 (6)	
H20	−0.3044	0.8049	0.9362	0.053*	
C21	−0.1359 (2)	0.8719 (2)	1.0397 (2)	0.0382 (5)	
C22	−0.0071 (2)	0.8519 (2)	1.0647 (2)	0.0360 (5)	
C23	0.0436 (2)	0.7436 (2)	0.9991 (2)	0.0376 (5)	
H23	0.1301	0.7296	1.0141	0.045*	
C24	−0.0552 (2)	1.0322 (2)	1.1871 (2)	0.0398 (5)	
C25	0.0438 (2)	0.9582 (2)	1.1613 (2)	0.0411 (5)	
H25	0.1280	0.9730	1.1986	0.049*	
C26	−0.0613 (3)	1.1537 (2)	1.2777 (2)	0.0434 (6)	
C27	0.5996 (3)	0.6981 (3)	0.6439 (3)	0.0676 (8)	
H27A	0.5659	0.6832	0.5592	0.101*	
H27B	0.6908	0.7113	0.6537	0.101*	
H27C	0.5628	0.7708	0.6899	0.101*	
Cl1	0.27357 (7)	0.52925 (6)	0.58111 (7)	0.0569 (2)	
N1	0.4448 (3)	−0.1220 (3)	−0.1192 (2)	0.0661 (7)	
N2	0.3540 (2)	−0.32906 (18)	0.3639 (2)	0.0475 (5)	
H2	0.3552	−0.4015	0.3784	0.057*	
N3	0.12773 (18)	0.34606 (16)	0.68429 (16)	0.0348 (4)	
H3A	0.1740	0.3935	0.6491	0.042*	
N4	0.00394 (18)	0.54081 (17)	0.84536 (17)	0.0382 (4)	
N5	−0.1755 (2)	1.20379 (19)	1.2756 (2)	0.0507 (5)	
H5A	−0.1858	1.2740	1.3258	0.061*	
H5B	−0.2388	1.1659	1.2240	0.061*	
O1	−0.16729 (16)	0.98325 (15)	1.11414 (15)	0.0439 (4)	
O2	0.0344 (2)	1.20077 (18)	1.34781 (18)	0.0616 (5)	
O3	0.5686 (2)	0.5922 (2)	0.6869 (2)	0.0730 (6)	
H3B	0.4921	0.5731	0.6673	0.110*	

### Atomic displacement parameters (Å^2^)

**Table:**

	*U*^11^	*U*^22^	*U*^33^	*U*^12^	*U*^13^	*U*^23^
C1	0.0437 (14)	0.0638 (17)	0.0454 (15)	0.0061 (12)	0.0123 (11)	0.0015 (13)
C2	0.0368 (12)	0.0535 (14)	0.0425 (13)	0.0042 (11)	0.0080 (10)	0.0039 (11)
C3	0.0483 (15)	0.0508 (15)	0.0524 (16)	0.0055 (12)	0.0119 (12)	−0.0086 (12)
C4	0.0533 (15)	0.0368 (12)	0.0602 (17)	0.0061 (11)	0.0097 (13)	−0.0011 (11)
C5	0.0392 (12)	0.0340 (11)	0.0506 (14)	0.0017 (10)	0.0045 (10)	0.0035 (10)
C6	0.0360 (12)	0.0348 (11)	0.0429 (13)	0.0047 (9)	0.0071 (10)	0.0050 (9)
C7	0.0415 (13)	0.0381 (12)	0.0445 (13)	0.0042 (10)	0.0064 (10)	0.0054 (10)
C8	0.0505 (14)	0.0387 (12)	0.0472 (14)	0.0044 (11)	0.0117 (11)	0.0087 (10)
C9	0.0420 (12)	0.0337 (11)	0.0427 (13)	0.0052 (10)	0.0092 (10)	0.0053 (9)
C10	0.0637 (16)	0.0337 (12)	0.0443 (14)	0.0110 (11)	0.0174 (12)	0.0077 (10)
C11	0.0580 (16)	0.0384 (12)	0.0439 (14)	0.0131 (11)	0.0079 (11)	0.0067 (10)
C12	0.0499 (15)	0.0416 (13)	0.0476 (14)	0.0096 (11)	0.0073 (11)	−0.0001 (11)
C13	0.0510 (14)	0.0320 (11)	0.0404 (13)	0.0038 (10)	0.0081 (11)	0.0009 (9)
C14	0.0455 (13)	0.0399 (12)	0.0325 (12)	0.0075 (10)	0.0036 (10)	0.0051 (9)
C15	0.0384 (12)	0.0412 (12)	0.0325 (11)	0.0080 (10)	0.0004 (9)	0.0024 (9)
C16	0.0430 (13)	0.0402 (12)	0.0355 (12)	0.0082 (10)	−0.0014 (10)	0.0031 (9)
C17	0.0409 (13)	0.0419 (12)	0.0391 (13)	0.0032 (10)	−0.0010 (10)	0.0008 (10)
C18	0.0354 (11)	0.0383 (11)	0.0327 (11)	0.0056 (9)	0.0051 (9)	0.0084 (9)
C19	0.0377 (12)	0.0437 (12)	0.0391 (13)	0.0030 (10)	0.0017 (10)	0.0033 (10)
C20	0.0338 (12)	0.0493 (13)	0.0471 (14)	0.0101 (10)	0.0031 (10)	0.0074 (11)
C21	0.0397 (12)	0.0361 (11)	0.0386 (12)	0.0087 (9)	0.0071 (10)	0.0077 (9)
C22	0.0366 (12)	0.0364 (11)	0.0353 (11)	0.0051 (9)	0.0076 (9)	0.0082 (9)
C23	0.0331 (11)	0.0399 (12)	0.0377 (12)	0.0050 (9)	0.0052 (9)	0.0059 (9)
C24	0.0466 (13)	0.0366 (11)	0.0347 (12)	0.0041 (10)	0.0045 (10)	0.0070 (9)
C25	0.0420 (13)	0.0401 (12)	0.0386 (12)	0.0056 (10)	0.0037 (10)	0.0060 (10)
C26	0.0579 (15)	0.0357 (12)	0.0367 (12)	0.0063 (11)	0.0100 (11)	0.0078 (10)
C27	0.083 (2)	0.0565 (17)	0.0645 (19)	0.0146 (16)	0.0063 (16)	0.0187 (14)
Cl1	0.0675 (5)	0.0391 (3)	0.0694 (5)	0.0037 (3)	0.0318 (4)	0.0125 (3)
N1	0.0635 (16)	0.0842 (18)	0.0510 (14)	0.0089 (13)	0.0171 (12)	0.0133 (13)
N2	0.0535 (13)	0.0309 (10)	0.0589 (13)	0.0042 (9)	0.0116 (10)	0.0109 (9)
N3	0.0399 (10)	0.0318 (9)	0.0324 (10)	0.0030 (8)	0.0082 (8)	0.0059 (7)
N4	0.0390 (10)	0.0381 (10)	0.0335 (10)	0.0061 (8)	0.0032 (8)	0.0026 (8)
N5	0.0554 (13)	0.0395 (11)	0.0523 (13)	0.0102 (10)	0.0117 (10)	0.0003 (9)
O1	0.0441 (9)	0.0389 (9)	0.0451 (10)	0.0120 (7)	0.0075 (7)	0.0032 (7)
O2	0.0724 (13)	0.0489 (11)	0.0529 (11)	0.0061 (10)	−0.0041 (10)	−0.0003 (9)
O3	0.0716 (14)	0.0594 (13)	0.0920 (17)	0.0092 (11)	0.0003 (13)	0.0315 (12)

### Geometric parameters (Å, º)

**Table:**

C1—N1	1.147 (4)	C15—H15B	0.9700
C1—C2	1.435 (4)	C16—N4	1.472 (3)
C2—C7	1.387 (3)	C16—C17	1.513 (3)
C2—C3	1.416 (4)	C16—H16A	0.9700
C3—C4	1.373 (4)	C16—H16B	0.9700
C3—H3	0.9300	C17—N3	1.495 (3)
C4—C5	1.393 (3)	C17—H17A	0.9700
C4—H4	0.9300	C17—H17B	0.9700
C5—N2	1.358 (3)	C18—C23	1.393 (3)
C5—C6	1.424 (3)	C18—N4	1.419 (3)
C6—C7	1.397 (3)	C18—C19	1.419 (3)
C6—C9	1.432 (3)	C19—C20	1.371 (3)
C7—H7	0.9300	C19—H19	0.9300
C8—C9	1.363 (3)	C20—C21	1.378 (3)
C8—N2	1.370 (3)	C20—H20	0.9300
C8—H8	0.9300	C21—O1	1.382 (3)
C9—C10	1.508 (3)	C21—C22	1.385 (3)
C10—C11	1.520 (3)	C22—C23	1.398 (3)
C10—H10A	0.9700	C22—C25	1.439 (3)
C10—H10B	0.9700	C23—H23	0.9300
C11—C12	1.531 (3)	C24—C25	1.352 (3)
C11—H11A	0.9700	C24—O1	1.377 (3)
C11—H11B	0.9700	C24—C26	1.484 (3)
C12—C13	1.515 (4)	C25—H25	0.9300
C12—H12A	0.9700	C26—O2	1.227 (3)
C12—H12B	0.9700	C26—N5	1.333 (3)
C13—N3	1.502 (3)	C27—O3	1.431 (3)
C13—H13A	0.9700	C27—H27A	0.9600
C13—H13B	0.9700	C27—H27B	0.9600
C14—N3	1.493 (3)	C27—H27C	0.9600
C14—C15	1.512 (3)	N2—H2	0.8600
C14—H14A	0.9700	N3—H3A	0.9100
C14—H14B	0.9700	N5—H5A	0.8600
C15—N4	1.459 (3)	N5—H5B	0.8600
C15—H15A	0.9700	O3—H3B	0.8200
			
N1—C1—C2	177.9 (3)	N4—C16—H16A	109.5
C7—C2—C3	121.5 (2)	C17—C16—H16A	109.5
C7—C2—C1	120.5 (2)	N4—C16—H16B	109.5
C3—C2—C1	117.9 (2)	C17—C16—H16B	109.5
C4—C3—C2	120.7 (2)	H16A—C16—H16B	108.1
C4—C3—H3	119.6	N3—C17—C16	111.14 (18)
C2—C3—H3	119.6	N3—C17—H17A	109.4
C3—C4—C5	117.8 (2)	C16—C17—H17A	109.4
C3—C4—H4	121.1	N3—C17—H17B	109.4
C5—C4—H4	121.1	C16—C17—H17B	109.4
N2—C5—C4	129.9 (2)	H17A—C17—H17B	108.0
N2—C5—C6	107.5 (2)	C23—C18—N4	123.0 (2)
C4—C5—C6	122.6 (2)	C23—C18—C19	118.9 (2)
C7—C6—C5	118.6 (2)	N4—C18—C19	118.0 (2)
C7—C6—C9	134.4 (2)	C20—C19—C18	122.4 (2)
C5—C6—C9	107.0 (2)	C20—C19—H19	118.8
C2—C7—C6	118.8 (2)	C18—C19—H19	118.8
C2—C7—H7	120.6	C19—C20—C21	117.1 (2)
C6—C7—H7	120.6	C19—C20—H20	121.5
C9—C8—N2	111.1 (2)	C21—C20—H20	121.5
C9—C8—H8	124.5	C20—C21—O1	126.2 (2)
N2—C8—H8	124.5	C20—C21—C22	123.0 (2)
C8—C9—C6	105.7 (2)	O1—C21—C22	110.7 (2)
C8—C9—C10	127.3 (2)	C21—C22—C23	119.5 (2)
C6—C9—C10	127.0 (2)	C21—C22—C25	105.5 (2)
C9—C10—C11	113.6 (2)	C23—C22—C25	134.9 (2)
C9—C10—H10A	108.8	C18—C23—C22	119.1 (2)
C11—C10—H10A	108.8	C18—C23—H23	120.5
C9—C10—H10B	108.8	C22—C23—H23	120.5
C11—C10—H10B	108.8	C25—C24—O1	112.0 (2)
H10A—C10—H10B	107.7	C25—C24—C26	131.2 (2)
C10—C11—C12	114.8 (2)	O1—C24—C26	116.9 (2)
C10—C11—H11A	108.6	C24—C25—C22	106.4 (2)
C12—C11—H11A	108.6	C24—C25—H25	126.8
C10—C11—H11B	108.6	C22—C25—H25	126.8
C12—C11—H11B	108.6	O2—C26—N5	124.6 (2)
H11A—C11—H11B	107.6	O2—C26—C24	120.2 (2)
C13—C12—C11	111.4 (2)	N5—C26—C24	115.2 (2)
C13—C12—H12A	109.3	O3—C27—H27A	109.5
C11—C12—H12A	109.3	O3—C27—H27B	109.5
C13—C12—H12B	109.3	H27A—C27—H27B	109.5
C11—C12—H12B	109.3	O3—C27—H27C	109.5
H12A—C12—H12B	108.0	H27A—C27—H27C	109.5
N3—C13—C12	114.26 (19)	H27B—C27—H27C	109.5
N3—C13—H13A	108.7	C5—N2—C8	108.8 (2)
C12—C13—H13A	108.7	C5—N2—H2	125.6
N3—C13—H13B	108.7	C8—N2—H2	125.6
C12—C13—H13B	108.7	C14—N3—C17	109.46 (17)
H13A—C13—H13B	107.6	C14—N3—C13	112.72 (17)
N3—C14—C15	112.17 (18)	C17—N3—C13	110.61 (17)
N3—C14—H14A	109.2	C14—N3—H3A	108.0
C15—C14—H14A	109.2	C17—N3—H3A	108.0
N3—C14—H14B	109.2	C13—N3—H3A	108.0
C15—C14—H14B	109.2	C18—N4—C15	115.32 (18)
H14A—C14—H14B	107.9	C18—N4—C16	116.13 (18)
N4—C15—C14	111.40 (19)	C15—N4—C16	110.43 (18)
N4—C15—H15A	109.3	C26—N5—H5A	120.0
C14—C15—H15A	109.3	C26—N5—H5B	120.0
N4—C15—H15B	109.3	H5A—N5—H5B	120.0
C14—C15—H15B	109.3	C24—O1—C21	105.34 (17)
H15A—C15—H15B	108.0	C27—O3—H3B	109.5
N4—C16—C17	110.67 (19)		
			
C7—C2—C3—C4	0.4 (4)	O1—C21—C22—C25	0.1 (3)
C1—C2—C3—C4	−177.9 (3)	N4—C18—C23—C22	−175.9 (2)
C2—C3—C4—C5	0.2 (4)	C19—C18—C23—C22	0.5 (3)
C3—C4—C5—N2	−179.1 (3)	C21—C22—C23—C18	−1.0 (3)
C3—C4—C5—C6	−0.4 (4)	C25—C22—C23—C18	178.5 (2)
N2—C5—C6—C7	178.9 (2)	O1—C24—C25—C22	−0.5 (3)
C4—C5—C6—C7	−0.1 (4)	C26—C24—C25—C22	180.0 (2)
N2—C5—C6—C9	0.1 (3)	C21—C22—C25—C24	0.2 (3)
C4—C5—C6—C9	−178.9 (2)	C23—C22—C25—C24	−179.4 (2)
C3—C2—C7—C6	−0.9 (4)	C25—C24—C26—O2	−2.5 (4)
C1—C2—C7—C6	177.4 (2)	O1—C24—C26—O2	178.0 (2)
C5—C6—C7—C2	0.8 (3)	C25—C24—C26—N5	177.7 (2)
C9—C6—C7—C2	179.1 (2)	O1—C24—C26—N5	−1.8 (3)
N2—C8—C9—C6	−0.6 (3)	C4—C5—N2—C8	178.4 (3)
N2—C8—C9—C10	−178.4 (2)	C6—C5—N2—C8	−0.5 (3)
C7—C6—C9—C8	−178.2 (3)	C9—C8—N2—C5	0.7 (3)
C5—C6—C9—C8	0.3 (3)	C15—C14—N3—C17	−53.8 (2)
C7—C6—C9—C10	−0.5 (4)	C15—C14—N3—C13	−177.34 (19)
C5—C6—C9—C10	178.1 (2)	C16—C17—N3—C14	55.1 (2)
C8—C9—C10—C11	7.9 (4)	C16—C17—N3—C13	179.89 (19)
C6—C9—C10—C11	−169.3 (2)	C12—C13—N3—C14	−58.5 (3)
C9—C10—C11—C12	179.2 (2)	C12—C13—N3—C17	178.6 (2)
C10—C11—C12—C13	80.7 (3)	C23—C18—N4—C15	−1.8 (3)
C11—C12—C13—N3	−177.70 (19)	C19—C18—N4—C15	−178.3 (2)
N3—C14—C15—N4	55.5 (3)	C23—C18—N4—C16	−133.3 (2)
N4—C16—C17—N3	−58.2 (3)	C19—C18—N4—C16	50.3 (3)
C23—C18—C19—C20	0.9 (4)	C14—C15—N4—C18	168.90 (18)
N4—C18—C19—C20	177.5 (2)	C14—C15—N4—C16	−57.0 (2)
C18—C19—C20—C21	−1.6 (4)	C17—C16—N4—C18	−167.80 (19)
C19—C20—C21—O1	−178.4 (2)	C17—C16—N4—C15	58.5 (2)
C19—C20—C21—C22	1.0 (4)	C25—C24—O1—C21	0.6 (3)
C20—C21—C22—C23	0.3 (4)	C26—C24—O1—C21	−179.84 (19)
O1—C21—C22—C23	179.77 (19)	C20—C21—O1—C24	179.0 (2)
C20—C21—C22—C25	−179.3 (2)	C22—C21—O1—C24	−0.4 (2)

### Hydrogen-bond geometry (Å, º)

Cg5 is the centroid of the C18–C22 ring.

**Table:**

*D*—H···*A*	*D*—H	H···*A*	*D*···*A*	*D*—H···*A*
N2—H2···O3^i^	0.86	2.25	2.971 (3)	141
N3—H3*A*···Cl1	0.91	2.18	3.0787 (19)	172
N5—H5*A*···Cl1^ii^	0.86	2.42	3.250 (2)	162
N5—H5*B*···N1^iii^	0.86	2.33	3.151 (4)	160
O3—H3*B*···Cl1	0.82	2.38	3.195 (2)	171
C13—H13*A*···O2^iv^	0.97	2.33	3.272 (3)	164
C19—H19···O3^v^	0.93	2.55	3.348 (3)	144
C14—H14*B*···*Cg*5^vi^	0.97	2.48	3.393 (2)	156

Symmetry codes: (i) −*x*+1, −*y*, −*z*+1; (ii) −*x*, −*y*+2, −*z*+2; (iii) −*x*, −*y*+1, −*z*+1; (iv) *x*, *y*−1, *z*−1; (v) *x*−1, *y*, *z*; (vi) −*x*, −*y*+1, −*z*+2.
